# Emotion displays in media: a comparison between American, Romanian, and Turkish children's storybooks

**DOI:** 10.3389/fpsyg.2014.00600

**Published:** 2014-06-17

**Authors:** Briana Vander Wege, Mayra L. Sánchez González, Wolfgang Friedlmeier, Linda M. Mihalca, Erica Goodrich, Feyza Corapci

**Affiliations:** ^1^Psychology, Grand Valley State UniversityAllendale, MI, USA; ^2^Department of Educational Psychology, Texas A&M UniversityCollege Station, TX, USA; ^3^Department of Psychology, Babes-Bolyai UniversityCluj, Romania; ^4^Department of Psychology, Bogazici UniversityIstanbul, Turkey

**Keywords:** emotion, children's storybooks, cross-cultural comparison, emotion norms, ingroup-outgroup comparison

## Abstract

Children's books may provide an important resource of culturally appropriate emotions. This study investigates emotion displays in children's storybooks for preschoolers from Romania, Turkey, and the US in order to analyze cultural norms of emotions. We derived some hypotheses by referring to cross-cultural studies about emotion and emotion socialization. For such media analyses, the frequency rate of certain emotion displays can be seen as an indicator for the salience of the specific emotion. We expected that all children's storybooks would highlight dominantly positive emotions and that US children's storybooks would display negative powerful emotions (e.g., anger) more often and negative powerless emotions (e.g., sadness) less often than Turkish and Romanian storybooks. We also predicted that the positive and negative powerful emotion expressions would be more intense in the US storybooks compared to the other storybooks. Finally, we expected that social context (ingroup/outgroup) may affect the intensity emotion displays more in Turkish and Romanian storybooks compared to US storybooks. Illustrations in 30 popular children's storybooks (10 for each cultural group) were coded. Results mostly confirmed the hypotheses but also pointed to differences between Romanian and Turkish storybooks. Overall, the study supports the conclusion that culture-specific emotion norms are reflected in media to which young children are exposed.

## Introduction

### Emotion displays in media: a comparison between Romanian, Turkish, and Euro-American children's storybooks

The study of emotion norms is important to better describe and explain cultural differences in emotion socialization. For example, experiencing positive emotions may be desirable universally. However, the quality of the experience may vary across cultures. Tsai (2007) demonstrated that Taiwanese Chinese rather prefer low-arousal positive emotions like contentment, whereas European Americans prefer high-arousal positive emotions like excitement; i.e., these two cultural groups differ in the desired (ideal) positive affect. This cultural difference in ideal positive affect was also reflected in children's storybooks of the two cultures (Tsai et al., 2007). These findings suggest that cultural artifacts, such as children's books, act as one of the specific pathways through which emotion norms are presented and can be culturally transmitted and learned. Therefore, children's storybooks may provide an important resource for children to learn about culturally appropriate emotions.

This study aims to analyze emotion norms as represented by emotion displays in children's storybooks for preschoolers and to expand the study of Tsai et al. (2007) in two ways. First, we aim to expand the cultural variation. Tsai et al. (2007) focused on the comparison of American and East Asian cultures, like many other studies. We aim to compare emotion displays in children's books from two Eastern European countries (Romania, Turkey) with books from the US. Second, Tsai et al. (2007) focused exclusively on the positive ideal affect. In the present study, we further include comparisons involving the displays of negative emotions as well as social context (ingroup/outgroup) in which emotion displays occur.

Studies about emotion norms are still scarce, but indirect evidence for potential cultural differences can be concluded from two different areas of research: (1) emotion display rules, emotion expression, and ideal affect that were studied in cross-cultural perspective over the last 10 years and, (2) studies about emotion socialization in different cultures that investigated parental beliefs about children's emotion competence. Both areas give some direct and indirect insight into cultural emotion norms.

## General cultural differences in emotion expression norms

Correlational studies analyzing the relations between emotion display rules and country characteristics showed that adults in more individualist countries endorse emotion expression overall more strongly than collectivistic countries (Matsumoto et al., 2008). The meta-analysis of emotion and culture by Van Hemert et al. (2007), which included 190 studies from 29 different countries, confirmed this result and also found that the same relation applies for the level of democracy and stability: countries with a higher level of democracy, higher observance of human rights and higher stability allow more emotion expression in general. The hypothesis that countries with higher uncertainty avoidance (i.e., societies with low tolerance for ambiguity and strict rules of belief and behavior; Hofstede, 2011) allow more negative emotion expressions could not be confirmed (Van Hemert et al., 2007).

Growing evidence with children also shows that school-aged children from East Asia (e.g., China, Korea), South Asia (e.g., Nepal, India), and the Middle East (e.g., Iran, Morocco) are less likely to be confrontational and more likely to hide their emotions, particularly anger, than children from the US or Western Europe (Cole et al., 2002; Novin et al., 2009, 2010, 2011; Wilson et al., 2012).

These differences in emotion expression at the country-level have typically been explained by differences in the self-construal dimension. The relative salience of the independent vs. interdependent self-construal has been conceptualized as playing a differential role in the organization of one's emotions, cognitions, and behaviors (Markus and Kitayama, 2010). Individuals in countries endorsing norms like individualism, equality, and autonomy show higher endorsement of independent self-construal, i.e., they perceive themselves as separate from others and unique, whereas individuals in countries endorsing collectivism, hierarchy, and embeddedness display higher interdependent self-construal, i.e., they perceive themselves as related to others.

The cultural emotion norms delineated in the adult emotion display rules literature are in line with emotion socialization studies in cross-cultural psychology. European American mothers elaborate and provide more detail when talking about emotions, and imbue their interactions with smiles and laughing to a greater extent than mothers from Japan, Indonesia, and China (see Fivush et al., 2006, and Tsai, 2007 for reviews). These findings imply that caregivers' culturally valued self-construal also acts to shape their emotion-related goals and socialization practices. In other words, emotion norms are learned and transmitted in line with the cultural models of emotion competence, defined as caregivers' expectations for children's understanding and expression of emotions. Research suggests that European-Americans favor an “individualistic” model of emotion competence, i.e., encouraging emotion expression in a more open way as parents strive to promote children's self-sufficiency, sense of autonomy and independence through creativity, assertiveness, and self-esteem (Greenfield et al., 2003). Consequently, self-expression and open communication of “ego-focused” emotions, such as anger, pride, and disgust, which support the assertion of the autonomous self are favored, while shame expression which may signal a threat to a child's self-esteem is discouraged (Markus and Kitayama, 2001).

Parents in collectivistic countries favor a relational model of emotion competence. In the relational model, proper behavior is prioritized into hierarchical relationships, such as respect for elders and loyalty to family, social harmony, and group interests (Matsumoto, 1991). Thus emotions that are ego-focused are considered potentially disrupting to interpersonal relations and are strictly controlled (Wang, 2003). In these societies, caregivers tend to promote emotion display rules which underscore the importance of interpersonal sensitivity and cultivate “other-focused” emotions like sympathy and shame in order to foster relational emotional competence (Chan et al., 2009).

## Cultural differences in emotion norms in Romania, Turkey and the US

Most cross-cultural studies focus on US-Asian comparisons with an emphasis on cultural differences in individualism and collectivism, whereas East European countries were not often included in cross-cultural studies. Investigating East European countries can contribute to a better understanding of the variability within collectivist and group-oriented countries. East European countries like Turkey and Romania are less individualistic than US but also less collectivistic than East Asian countries like China or South Korea (Hofstede, 2001). Both countries have higher uncertainty avoidance, i.e., lower tolerance for ambiguity, than the US and East Asian countries (Hofstede, 2001). Romania and Turkey are qualified as countries that endorse the values of hierarchy (in opposition to egalitarianism) and embeddedness (in opposition to autonomy) in contrast to US (Schwartz, 2008). These similar value profiles make them also distinct from East Asian countries that endorse those values much more strongly.

Confirming these expectations, Turkish student samples did not endorse emotion expression as much as Americans did, but more compared to Hong Kong students in the emotion display rule studies mentioned above (Matsumoto et al., 2008). A qualitative cross-cultural study (Denham et al., 2004) about American, Romanian, and Japanese parents' strategies to manage their children's emotions highlighted Japanese parents' stress on restraining their preschoolers' emotion displays or downplaying their means of emotion expression in social situations. In contrast, Romanian parents did not mention such restrictions about emotion expression, and were similar to American parents' answers, but—in contrast to the American mothers—they were also eager to rather avoid the exposure of their child to negative emotions including showing their own negative emotion (e.g., anger; see also Bassett et al., unpublished manuscript). Finally, evidence from research that pertains to emotion socialization shows that Turkish mothers were more likely to make appeals to others' behavioral approval and feelings compared to US mothers in discipline contexts (Catay et al., 2008), pointing to Turkish mothers' efforts to cultivate relational emotion competence by prioritizing appropriate conduct and sensitivity to others' emotions. Altogether these studies support the general claim that emotion norms in Turkey and Romania are not identical with norms in East Asian countries, but they also diverge from American norms.

Romania is rarely included in multinational studies, but due to the common value orientations, we expect similarities in emotion norms between these two countries. In light of limited recent research on emotion display rules and emotion-related parenting, and considering the common value orientations in Turkey and Romania (Kagitcibasi, 2007; Friedlmeier and Gavreliuc, 2013) that emphasize respect, self-restraint, and harmonious relations, we predicted higher intensity of overall emotion expressions in American storybooks compared to the Romanian and Turkish storybooks for preschoolers.

Cultural differences in emotion endorsement do not only refer to the intensity of the expression but also to the frequency of their display in the media. As this study investigates images in children's storybooks, an often repeated display of a distinct emotion can be interpreted as a salient feature of a specific culture. This means that this emotion is considered important and relevant, while a low rate of displays rather points to low desirability of such an emotion.

## Cultural differences in the valence of emotions

Besides the generally stronger endorsement of emotion expression in the US, a differentiation along the valence of emotions is important as norms for positive and negative emotions may vary across cultures. Diener and Lucas (2004) analyzed the desired emotions for children by asking students in 48 different countries to rate the statement “I hope my daughter will be happy” on a 9-point scale from 1(*strongly disagree*) to 9 (*strongly agree*). Respondents in all countries desired high levels of happiness for their children. Happiness as desired for their children was more strongly endorsed by American than by Turkish students, but the difference was not significant (Diener and Lucas, 2004). This pattern suggests that adults in all cultures aim mostly to encourage positive emotions in young children (Cole and Tan, 2006). Therefore, we expected that children's storybooks from all three countries would show displays of positive emotions in a dominant way compared to negative emotions.

Despite the predominant display of positive emotions across cultural groups, the displays of positive emotions appear to differ in the intensity and activity level as a function of emotional norms in each culture. Tsai et al. (2007) compared the affective content of the best-selling storybooks in the US and Taiwan. Although the best-selling children's storybooks from the United States (US) and Taiwan did not differ in the number of pictures with positive affective states, books from the US contained significantly more pictures of characters depicted with excited expressions (i.e., wider smiles, laughing) than those in the Taiwanese storybooks. The emphasis of contentment and quiet activity may be less salient in Eastern European countries such as Romania and Turkey. However, based on the positive relation between individualism and expression norms for happiness (Matsumoto et al., 2008), strong positive expression may be more of a norm in the US compared to Turkey and Romania given the stronger collectivistic orientation of these countries. Hence, we expected a higher intensity of positive emotion displays in the American books compared to the Turkish and Romanian books.

Most studies to date treated negative emotions as a unitary construct without considering their valence (Van Hemert et al., 2007). In doing this, the researchers missed the point that there are two opposite classes of emotions within the negative emotions in regard to action readiness. Timmers et al. (1998) introduced the terms “powerless” and “powerful negative emotions” when studying gender differences in emotion expression, and Halberstadt (1986) called them “dominant” and “submissive negative emotions.” Kitayama and colleagues have also made a differentiation between socially engaging and disengaging negative emotions based on the emotion themes grounded in independence or interdependence (Kitayama et al., 2006). Powerless negative, socially engaging, or submissive negative emotions, like sadness, fear, and shame, include the action readiness to withdraw, flee, or hide, and such emotions call for emotional support by others. Powerful negative, socially disengaging, or dominant negative emotions, like anger, jealousy, contempt, and frustration, include the action readiness to attack, to hurt, or to offend, and such emotions rather represent a threat to others. We will use the terms “powerful negative” and “powerless negative emotions” for this article.

These distinctions are being increasingly employed, and the differentiation between powerful and powerless negative emotions is essential for cultural comparisons (Kitayama et al., 2006). Emotions have both intra- and interpersonal meanings, which serve different functions in specific cultures (Matsumoto et al., 2008). Individualistic cultures place a higher emphasis on intrapersonal meanings, because they foster expressivity and place the individual at a higher level of importance compared to social relationships (Matsumoto et al., 2008). In collectivist cultures, powerless negative emotions are well accepted since they do not bring any harm to the group. However, powerful negative emotions are seen as disruptive and a threat to the group, and the need to suppress them is seen as much more important compared to individualistic cultures.

Supporting this view, Americans reported the intensity of experiencing powerful negative emotions (e.g., anger) as stronger than Japanese in response to negative events, while the reverse pattern was found for powerless negative emotions (Kitayama et al., 2006). In the study about desired emotions for their children, Diener and Lucas (2004) asked students in regard to anger (“I hope my daughter will not express anger, even when she has a reason for doing so”). Turkish students desired higher anger suppression for their children compared to American students.

Emotion socialization research also reveals that mothers from Turkey (Corapci, 2012; Corapci et al., 2012) and India (Raval and Martini, 2009) were more likely to encourage the expression of sadness compared to anger. Indian mothers also reported less scaffolding and more minimization for child anger than sadness (Raval and Martini, 2009). Unlike Indian caregivers, US caregivers were observed to give more attention to children's (4–6 years) anger than sadness and anxiety, reflecting differential parental socialization pressure to different child emotions (Chaplin et al., 2005).

Taken together, emerging research evidence supports the view that the expression of negative powerful emotions are encouraged more in individualistic cultures where relatively greater importance is placed on the independent self and the communication of self-focused; even interpersonally disengaging emotions is seen as a way to foster autonomy and assertiveness. Conversely, the experience and expression of negative powerless emotions are discouraged in Western cultures, which may signal a threat to one's self-esteem. Therefore, we predicted that American children's storybooks would display powerful negative emotions more often and powerless negative emotions less often than the Turkish and Romanian books. These cultural differences in powerful and powerless negative emotions may not only have implications for the frequency of these displays in books but also to the presented intensity of the expressions. We expect that American storybooks will display a higher expression intensity for powerful negative expressions and lower intensity for negative powerless emotions than Turkish and Romanian books.

## Cultural differences of emotions as a function of social context

Emotion norms not only vary across cultures in regard to the overall expression endorsement and the valence of the emotion but also as a function of social context. The emotion display rules study by Matsumoto et al. (2008) showed that members of individualist cultures endorsed stronger expression of negative emotions and weaker expression of positive emotions toward ingroup members (i.e., familiar social partners like family members and friends) compared to outgroup members (i.e., less familiar social partners like colleagues and acquaintances). The correlations were strong for negative powerful emotions (anger, contempt, disgust) and less strong but still significant for negative powerless emotions (sadness and fear). On the other hand, students in more collectivist countries endorsed that negative emotions, especially negative powerful emotions, should be expressed more intensely toward outgroup rather than ingroup members. Research with children also revealed that Indian, Korean, and Iranian children hide their anger more in the presence of their parents than their peers, while the reverse pattern has been documented for US and Dutch children (Raval et al., 2007; Novin et al., 2009, 2010). These findings fit with the values of collectivistic cultures, where an intense expression of negative powerful emotions toward ingroup members should be avoided strongly as group cohesion is threatened by this.

Collectivistic cultures also foster higher differentiation between ingroup and outgroup relationships compared to individualistic cultures (Triandis, 1995; Kashima and Kashima, 2003). A comparison of emotion display norms for negative powerful emotions between Canadian, American, and Japanese students confirmed this norm difference (Safdar et al., 2009). Japanese students showed the strongest distinction between ingroup and outgroup and evaluated it as more appropriate to express negative powerful emotions more intensely toward outgroup than toward ingroup members (Safdar et al., 2009). In light of research conducted with adults and children, we expected that the frequency and intensity of emotion expressions will differ more strongly for ingroup-outgroup contexts in Romanian and Turkish storybooks compared to American storybooks.

## Coding of emotions in storybooks

Beside the theoretical framework, some methodological remarks are necessary as this study does not test individuals but refers to coding and analyzing illustrations of emotion expressions in children's storybooks. According to the Facial Action Coding System (FACS; Ekman and Friesen, 1978), emotion expressions can be analyzed as combinations of specific action units which refer to contractions of specific facial muscles (i.e., raised inner eyebrow, lips parted, nose wrinkled). Tsai et al. (2007) used part of this system to identify facial patterns of positive expression in storybooks. This analytical coding is considered a more objective measure compared to a synthesized decoding of an emotion that requests a synthesis of mimic (facial features), posture, gesture, causes, and context by the coder. However, the analytical approach is limited in several respects. First, the facial features of FACS are only determined for six distinct emotions (happiness, sadness, fear, anger, surprise, disgust), and do not include emotions like jealousy, shame, embarrassment, contempt, pride, and others. Second, the facial displays in the books are somewhat ambiguous as illustrators use different ways to draw the facial expressions. Additional information through gestures and postures can help determine the type of expressed emotion. Furthermore, illustrators from different cultures may use different features to determine emotion expression drawings. Cross-cultural research has shown that Japanese use the positions and form of the eyes to decode sadness and joy, whereas Americans focus on the mouth region, and this difference is also reflected in emoticons, combinations of keys that combine to form an approximate facial expression used in electronic communication (Yuki et al., 2005). Therefore, a synthesized judgment based on mimic, posture, gesture, and context about the emotion and the intensity of expression was chosen as a viable and appropriate way for coding these illustrations.

The analyses focus on the frequency rates as well as intensity of emotion expressions. As mentioned above, a frequent display of the same emotion can be interpreted as conveying high relevance of such emotion to the audience whereas the lack thereof may point to the fact that such an emotion is rather downplayed and seen as irrelevant.

## Hypotheses

To summarize, this study aims to analyze the emotion norms by examining the images in storybooks for preschoolers from the US, Romania, and Turkey. Using the cultural models of self-construal (Markus and Kitayama, 2001) and emotion competence (Chan et al., 2009) as guiding theoretical frameworks and building on previous cross-cultural research on emotion display rules and emotion socialization, we predicted four main hypotheses:

1. We expected that the storybooks from all three countries would display positive emotion displays to a greater extent than negative emotions.
2. Negative emotion displays in American children's storybooks will represent a higher proportion of negative powerful and a lower proportion of negative powerless emotion expressions compared to Turkish and Romanian books.
3. American children's storybooks will display higher intensity of expressivity for positive and powerful negative emotions compared to both Romanian and Turkish books. Due to the limited evidence, the examination of the cultural differences in negative powerless emotions is exploratory in the current study.
4. Emotion expression frequency and intensity toward ingroup vs. outgroup members will differ more strongly in the Romanian and Turkish storybooks compared to American storybooks, particularly for powerful-negative emotions in the light of previous research.

## Methods

### Selection of books

We sampled 10 American^1^, 10 Romanian and 10 Turkish children's storybooks for preschoolers or younger. Following the selection criteria used in previous research (Tsai et al., 2007), the most popular books in the U.S. were identified through online resources such as Amazon.com and through bookstores' bestseller lists in Turkey and Romania (see Table 1). The author and/or the illustrator are not necessarily members of the respective culture as some Turkish and Romanian books are translations from German or English books. Since these are best-selling storybooks, they are widely distributed and many young children are most likely to be exposed to these books. Beside popularity, the second selection criterion referred to the evaluation of the characters' emotion expression. Based on the most relevant mimic features according to Ekman et al. (1972), distinctive eyes, eyebrows, and mouth were necessary criteria. Books with animals as the main characters were acceptable as long as the facial features were human like enough to code. A third selection criterion was the requirement that the books had to have a storyline and a protagonist. This last criterion was deemed necessary because emotions are not static but rather dynamic; i.e., emotions (experience and expression) are evoked by a cause and also include action readiness to act upon the experienced emotion. Books with a storyline present emotions not just in static displays but give action-related information (e.g., cause of the emotion and action consequences) which convey information about emotions in a more vivid and accurate way. Taken together, these three selection criteria of (1) being widely distributed, (2) showing codable features, and (3) sharing a storyline served as basis of equivalence for all books.

**Table 1:** **Book titles of the selected books**.

**Children's storybooks from**		
**US**	**Turkey**	**Romania**
Alexander and the Terrible, Horrible, No good, Very Bad Day (2009)^a^	Ben Bir Ressamım! Dali (I am a painter! Dali) (2010)	Bobiţă şi Buburuză La Grădiniţă: Păianjenul din peşteră (Bobiţă and ladybug in kindergarden: spider from the cave (2012)^i^
Love you forever (2011)^b^	Hayvanları Çoook Seviyorum! Veli (I love animals! Veli) (2010)	Pupici pentru tatici (Kisses for daddies)(2008)^j^
I love You, Stinky face (1997)	Bir çizgi film Daha (One more cartoon) (2010)	Pinocchio (Pinocchio) (2011)^k^
The Potty Book for Girls (2000)	Değnek Adam (Stick man) (2008)^c^	Sora-cea-mica (Small sister) (2007)^l^
I'm a big Brother (1997)	Yavru Ahtapot Olmak Çok Zor (It is difficult to be a small octopus) (2011)^d^	Cenusareasa (Cinderella) (2009)^m^
Cloudy with a Chance of Meatballs (1978)	Sevim Ak Eskiler Alirim! (I buy old stuff!) (2007)	Gandaceii saritori (The jumping bugs) (2011)^n^
Max's Daddy goes to the hospital (1989)	Cemile oyuncaklarini paylasmak istemiyor (Camille does not want to lend her toys) (2006)^e^	Angelina şi Printesa (Angelina and the princess) (2006)^o^
No, David! (1998)	Ece ile Efe Hayvanat Bahcesinde (Zoe and Theo are in the zoo) (2008)^f^	Frumoasa şi Bestia (The beauty and the beast)(2011)^p^
David Goes to School (1999)	Atakan Cok Fazla Seker Yiyor (Atakan eats a lot of candy) (2007)^g^	Winnie de Pluş: De ce să dormi după-amiaza? (Winnie the Pooh and Friends: Why take a nap now?) (2011)^q^
Fancy Nancy (2006)	Zogi (A gold star for Zog) (2010)^h^	Alba –ca- Zapada (Snowhite) (2011)^r^

a Original published in 1972;

b Canadian Book, Original published in 1986;

c Original publish in English (UK; 2008);

d First published in 2008;

e Original published in French (2001);

f Original published in French (2002);

g Original published in French (2004);

h Original published in English (UK; 2010);

i Original published in Hungarian (2009);

j Original published in English (Australia; 2005);

k Original published in Italian (1883), adapted and translated in 2011;

l Original published in English (2007);

m Old European folk tale first published in French (1697);

n Original published in English (Australia; 2011);

o Original published in English (1984);

p Original published in French (1740); adapted and translated into Romanian language by Disney Enterprise;

q Original published in English (UK; 1926);

r *Old European folk tale first published in German (1812); adapted and translated in 2011*.

### Procedure

First, the main coder (coder A) identified all codable characters in a book and numbered them to ensure that all raters would code the same images in each book. Only the characters in the story whose faces were fully displayed were coded. The images on the covers and title pages were not coded. Then each character was coded for identification of gender, social context, type of distinct emotion and intensity of expression.

All coders were trained and familiarized with the Facial Action Coding System (FACS) to apply an analytical approach for coding. They received some training faces to achieve high interrater agreement before they started coding. Two American coders were involved: Coder A coded all books, coder B coded all American books and the majority of Romanian and Turkish books. A Romanian coder (coder C) coded all Romanian books, and a Turkish coder (coder D) rated some of the Turkish books in order to control for cultural bias. This coding strategy ensured that three ratings were available for each book and one of the coders was a member of the respective cultural group. For data analysis, the majority code of the three was used to create the final data set. In the rare cases that the three coders showed three different codes, the final decision was made by a fourth coder.

### Measures

#### Distinct emotion coding

Besides the facial expression of the character, the posture and gestures as well as contextual features like the description in the text or the drawn situation in the total picture allowed more reliable identification of the specific emotion. For this purpose, the Turkish and Romanian books were translated into English in order to facilitate the identification of the characters' distinct emotions. The American books were not translated as the Romanian and Turkish coders were fluent in English. The type of distinct emotion was coded as an overall evaluation of the displayed emotion by the rater, who considered facial expression and posture of a character and took into account contextual features including the text.

The list included 14 distinct emotions that were further classified into three types of emotions: *Positive emotions*, e.g., happiness, excitement, surprise, pride; *negative powerless emotions*, e.g., fear, sadness, embarrassment, shame, worry/anxiety; and *negative powerful emotions*, e.g., anger, disgust, dislike, contempt, jealousy. The interrater reliabilities for the emotion display variables across the four raters and the three countries were acceptable for the distinct emotion coding with a mean *Cohen's* κ = 0.89 ranging from 0.79 (rater A and D for Turkish books) to 0.96 (rater A and B for Romanian books). A frequency score was derived for each type of emotion based on the total number of counts across all books.

#### Intensity of expression coding

Intensity of the emotion expression was rated on a 4-point scale from 0—no expression, 1—weak expression, 2—medium expression, and 3—strong expression. The intraclass correlations for average measures across the respective three coders were 0.91 for American, 0.90 for Turkish, and 0.92 for Romanian books.

#### Social context coding

Social context code was differentiated into four categories. The protagonist was (1) with ingroup members, i.e., with familiar relatives (a parent, sibling or grandmother that lived in the house), best friend, teacher, significant other, (2) with outgroup members, i.e., with unfamiliar persons (e.g., strangers), (3) in a mixed group context, i.e., familiar and unfamiliar targets were present, or (4) alone. For hypothesis testing, only ingroup/outgroup context was analyzed. The alone context and mixed group were not considered further as no ingroup/outgroup distinctions can be made.

Each character was also coded for gender (male, female, or not known) and for representing the protagonist of the story (yes/no category). The interrater reliabilities for these variables were satisfactory with a mean κ = 0.90 for social context ranging from 0.84 (Rater B and Rater C for Turkish books) to 0.93 (Rater A and Rater C for Turkish books) and a mean κ = 0.98 for gender and protagonist ranging from 0.98 to 1.00.

## Results

### Cultural differences in the features of the storybook images

Preliminary analyses examined whether the American, Turkish, and Romanian storybook images were equivalent in terms of the quantity of images, the features of the characters and the contextual setting of the images. A total of 1118 images identified as fulfilling the criteria of fully displaying a character's facial and posture features were coded across the 30 storybooks. The number of coded images in Romanian books (*n* = 430) were significantly higher compared to the American books (*n* = 318), χ^2^_(1)_ = 16.77, *p* < 0.001 and Turkish books (*n* = 370), χ^2^_(1)_ = 4.50, *p* = 0.033, and the Turkish books had more figures than the American books, χ^2^_(1)_ = 3.93, *p* = 0.047. Since the number of the book pages did not vary across the three countries, *F*_(2, 27)_ = 2.59, *p* = 0.094, the higher numbers of overall images in the Romanian and Turkish books is due to the presence of more people displaying emotions in one scene.

**Figure 1:**
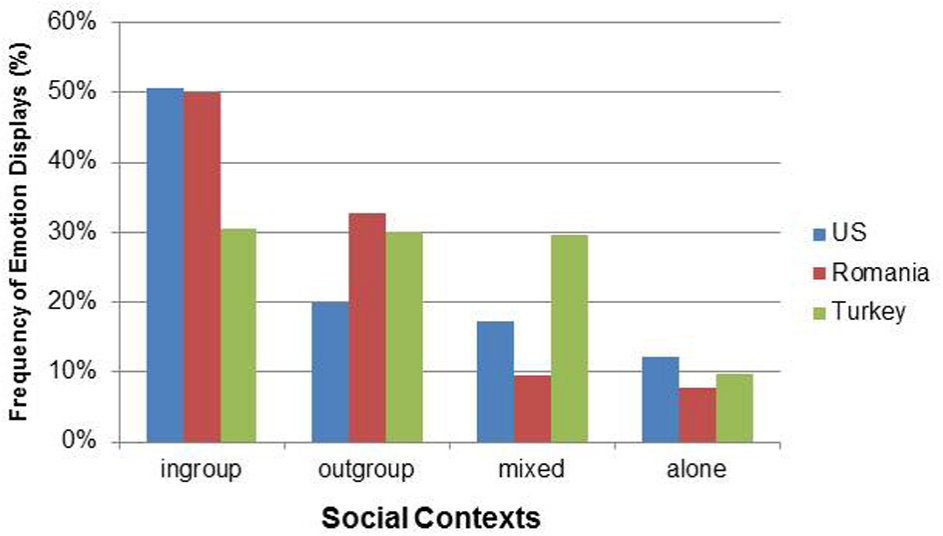
**Distribution of social contexts for the three cultural groups**.

The gender of the images did not vary significantly across cultures, χ^2^_(2)_ = 4.03, *p* = 0.133. The relative frequencies of displays in social contexts differed significantly across the three cultures, χ^2^_(6)_ = 81.94, *p* < 0.001 (see Figure 1). A main difference occurred for a direct comparison between ingroups and outgroups (by excluding mixed and alone): American books displayed emotions more in contexts with ingroup members, i.e., familiar persons (71.88%) compared to Romanian books (60.39%), χ^2^_(1)_ = 17.50, *p* < 0.001 and the Romanian proportion of displays to ingroup members was also significantly higher compared to the Turkish books (50.45%), χ^2^_(1)_ = 16.66, *p* < 0.001.

Many scenes in Turkish books represented a mixture of familiar and unfamiliar targets present at the same time (29.73%) which was significantly different from Romanian (9.53%), χ^2^_(1)_ = 43.56, *p* < 0.001, but not from the American books (17.30%), χ^2^_(1)_ = 0.01, *p* = 0.957. There was no cultural difference regarding displays in alone contexts, χ^2^_(2)_ = 4.36, *p* = 0.113, with an occurrence rate of about 10% (American books: 12.2%, Romanian books: 7.67%, and Turkish books: 9.73%; see Figure 1). No cultural differences occurred for protagonist, χ^2^_(2)_ = 0.59, *p* = 0.746: All books showed displays of the protagonist about 40% of the time, χ^2^_(2)_ = 0.04, *p* = 0.812.

### Descriptive results of emotion displays

The frequency distribution for different types of distinct emotions showed that among positive emotions, happiness was the dominant emotion in all three cultures (62.58%, *n* = 649) followed by surprise (7.52%, *n* = 78); interestingly, pride was rarely displayed (0.77%, *n* = 8) and never occurred in the Turkish books. Among negative powerless emotions, worry/anxiety was the most prevalent emotion in all three groups (12.25%, *n* = 127) followed by sadness (5.98%, *n* = 62). Guilt was only displayed one time in Turkish and Romanian books and never in American books. Embarrassment was coded only in Romanian books (0.48%, *n* = 5). Among negative powerful emotions, anger occurred more often (5.59%, *n* = 58) in all three cultural books compared to disgust (0.58%, *n* = 6), which never showed up in the Turkish books. Contempt or jealousy did not occur at all in any of these storybooks.

### Hypotheses testing

#### Emotion expression frequency

To determine cultural and contextual differences regarding the occurrence rates of emotions, loglinear analyses were computed. This statistical technique can be described as ANOVA for a categorical variable as dependent variable as it allows calculating the main effects and interaction effects of independent variables on the dependent variable. Specific group differences are computed as Maximum Likelihood chi-square tests. The unit of analysis is percentages of the three types of emotions (positive, negative powerless, and negative powerful emotions) across all books in each culture. First, we included gender as an independent variable. Since no interaction effects between gender and other independent variables occurred, we report the analyses without gender.

**Figure 2:**
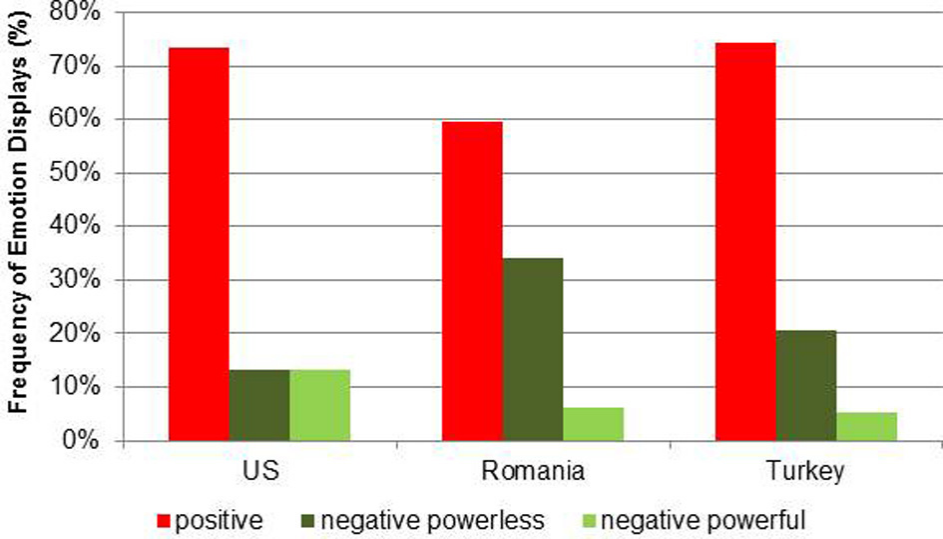
**Frequency rates of emotion displays in relation to cultural groups**.

The overall loglinear analysis for positive and negative emotion displays by cultural group showed a significant effect for type of emotions, χ^2^_(1)_ = 152.98, *p* < 0.001. Compared to negative emotions, positive emotions were significantly more frequently represented in the books of all three cultural groups (about 69% positive and 31% negative emotions (see Figure 1), confirming hypothesis 1. However, there were also cultural differences regarding the frequencies of positive emotions, χ^2^_(2)_ = 25.28, *p* < 0.001. Romanian books displayed proportionally less positive emotions (59.53%) compared to American books (73.58%), χ^2^_(1)_ = 4.07, *p* = 0.044, and to Turkish books (74.32%), χ^2^_(1)_ = 6.20, *p* < 0.013. The percentage of positive emotions in the Turkish and American books did not differ, χ^2^_(1)_ = 0.05, *p* = 0.826 (see Figure 2).

The additional analysis within the negative emotions (powerful vs. powerless negative emotions) yielded strong significant cultural group differences, χ^2^_(2)_ = 34.27, *p* < 0.001. American books displayed negative powerless and negative powerful emotions equally (50% resp. 50%), whereas the Romanian and Turkish books had a significantly higher percentage of negative powerless compared to negative powerful emotions (Turkey: 80.00%; MLχ^2(1)^ = 13.89, *p* < 0.001; Romania: 84.48%, MLχ^2^_(1)_ = 31.63, *p* < 0.001) (see Figure 2). The proportions of negative powerless and powerful emotions were similar for Romanian and Turkish books, MLχ^2^_(1)_ = 3.628, *p* = 0.07 (see Figure 2). These results confirm hypothesis 2.

Next, we explored the effects of social context on the occurrence of emotion displays. The loglinear analysis confirmed that the interaction effect for cultural group and social context was significant, LRχ^2^_(12)_ = 40.93, *p* < 0.001. We tested the interaction effect separately for each type of emotion.

**Figure 3:**
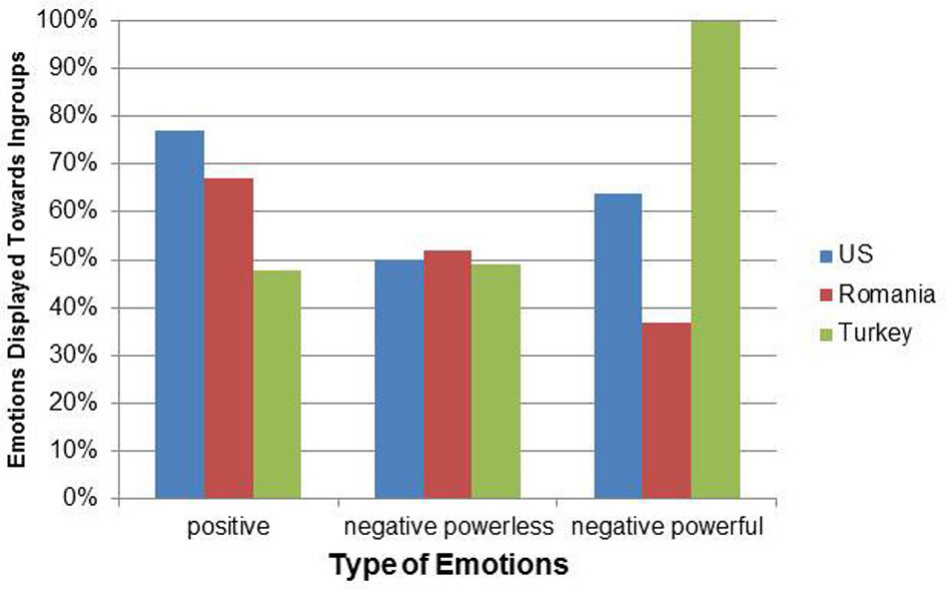
**Emotion displays toward ingroup members compared to outgroup members in relation to cultural groups and type of emotions**.

Positive emotions were displayed more toward ingroup (77.19%) than outgroup members in the American books compared to the Romanian books (67.13%), χ^2^_(1)_ = 17.56, *p* < 0.001. Turkish books were more balanced in displaying positive emotions (ingroup: 47.80%, outgroup: 52.20%) than the Romanian books χ^2^_(1)_ = 28.32, *p* < 0.001 (see Figure 3).

For negative powerless emotions, all books displayed the emotion in a balanced way across ingroup and outgroup (US: 50.00%; Turkey: 49.09%; Romania: 52.07% for ingroup), MLχ^2^_(1)_ = 0.15, *p* = 0.929) (see Figure 3).

Negative powerful emotions were displayed more often to ingroup members (63.89%) than to outgroup members (36.11%) in the American books and more to outgroup members (63.16%) than ingroup members (36.84%) in Romanian books. Turkish books showed an unexpected pattern as all negative powerful emotions were displayed toward ingroup members. However, none of these comparisons was significant (overall LR-χ^2^_(2)_ = 4.00, *p* = 0.135), because the occurrence rate of negative powerful emotion displays was very low in these books: *n* = 10 in Turkish books, *n* = 19 in Romanian books, and *n* = 36 in American books (see Figure 3).

### Emotion expression intensity

**Figure 4:**
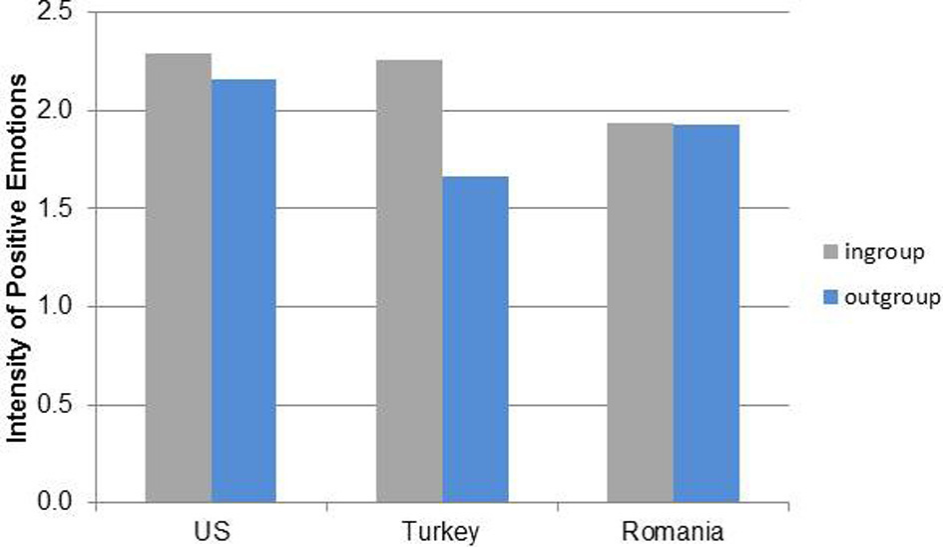
**Means of intensity of positive emotion expressions for cultural group and social context (ingroup/outgroup members)**.

To test hypotheses 3 and 4, we computed separate 3(culture) × 2(context) ANOVAs for each type of emotion (see Table 2). The ANOVA for positive emotions was significant with *r*^2^ = 0.088. Positive emotions were expressed more strongly in American books (*M* = 2.26, *SD* = 0.73) compared to both Romanian (*M* = 1.94, *SD* = 0.78) and Turkish books (*M* = 1.95, *SD* = 0.65), *F*_(2, 531)_ = 6.51, *p* = 0.002. Furthermore, the context effect was only significant for the Turkish books, *F*_(1, 153)_ = 35.33, *p* < 0.001, and not for American, *F*_(1, 160)_ = 0.85, *p* = 0.357 and Romanian books, *F*_(1, 214)_ = 0.02, *p* = 0.894. In Turkish books, positive emotions were expressed more strongly to ingroup members (*M* = 2.26, *SD* = 0.62) than outgroup members (*M* = 1.66, *SD* = 0.55) (see Table 2 and Figure 4).

**Table 2:** **Effects of cultural group and social context on emotion expression intensity**.

**Culture**	**US**			**Romania**		**Turkey**		***F*-values**
**Social context**	**In M (*SD*)**	**Out M (*SD*)**	**In M (*SD*)**		**Out M (*SD*)**	**In M (*SD*)**	**Out M (*SD*)**	
Positive emotions	2.29^a^ (0.71)	2.16^a^ (0.80)	1.94^b^ (0.73)		1.93^b^ (0.88)	2.26^a^ (0.62)	1.66^b^ (0.55)	A: 6.51^**^, B: 13.31^***^, C: 7.80^***^
Negative powerless emotions	2.62^a^ (0.51)	2.31^b^ (0.85)	2.41^a^ (0.59)		1.81^b^ (0.80)	2.15^a^ (0.53)	1.89^b^ (0.63)	A: 3.96^*^, B: 11.63^***^, C: 1.48

* p < 0.05;

** p < 0.01;

*** *p < 0.001*.

*In: Ingroup members; Out: Outgroup members; A: Main effect “Cultural group”; B: Main effect “Social context”; C: Interaction effect “Cultural group x social context.” Means with different superscripts differ significantly*.

**Figure 5:**
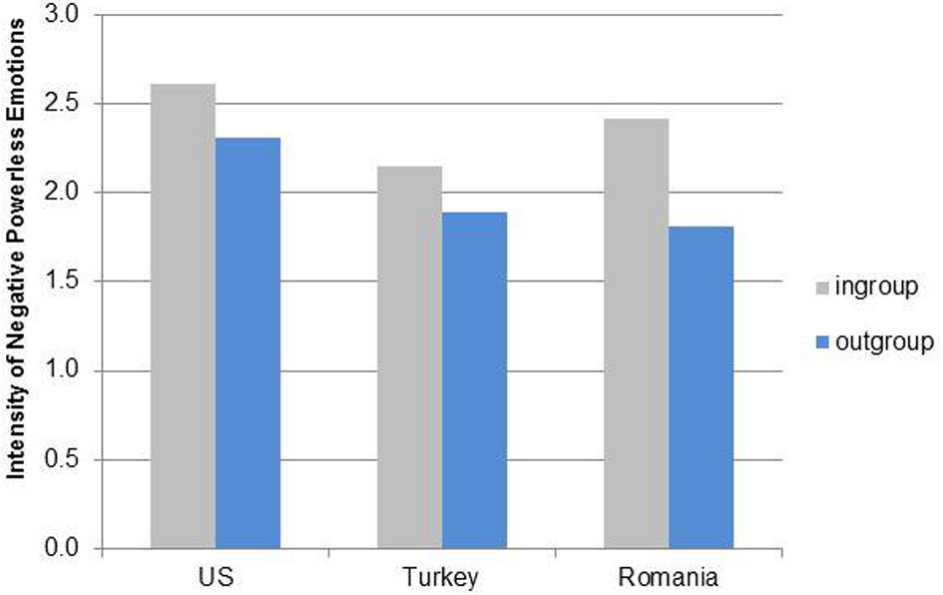
**Means of intensity of negative powerless emotion expressions for cultural group and social context (ingroup/outgroup members)**.

For negative powerless emotions, the effect size was *r*^2^ = 0.154. A similar cultural group effect as for positive emotions occurred, *F*_(2, 196)_ = 3.96, *p* = 0.021. Negative powerless emotions were expressed more strongly in the American books (*M* = 2.46, *SD* = 0.71) compared to the Romanian (*M* = 2.12, *SD* = 0.76) and Turkish books (*M* = 2.02, *SD* = 0.59). The context effect did not vary by country. Overall, negative powerless emotions were expressed more strongly toward ingroup members (*M* = 2.37, *SD* = 0.58) compared to outgroup members (*M* = 1.90, *SD* = 0.78), *F*_(1, 196)_ = 11.63, *p* < 0.001 (see Figure 5). No significant effects occurred for negative powerful emotions, *F*_(4, 60)_ = 1.50, *p* = 0.214, due to the low number of displays, especially in Romanian and Turkish books.

To summarize, hypothesis 3 was only confirmed for positive emotions. The hypothesis about ingroup/outgroup effects (hypothesis 4) with respect to emotion intensity was confirmed for American and Turkish books in regard to positive emotions but not for Romanian books.

## Discussion

This study aimed to investigate cultural differences of emotion displays in American, Turkish, and Romanian children's storybooks. In contrast to studies about emotion displays and emotion norms with participants, this study of fictitious displays in books allows analysis of frequencies as indicators for the importance of an emotion. Hence we expected that such media analysis would reflect commonalities and cultural differences in emotion norms between these different cultural groups. Going beyond the former studies that merely focused on positive emotions to investigate the cultural norms of emotion experience and expression in children's literature (Tsai, 2007), we investigated both positive and negative emotion displays in the storybooks. Based on the review of the current literature in this area, four hypotheses were formulated and tested.

### Dominance of positive emotions

First, the expectation that positive emotions would be more frequently displayed in all children's storybooks compared to negative emotions (hypothesis 1) was confirmed. This result is in line with caregivers' desires to see the experience of positive emotions across many cultures (Diener and Lucas, 2004) and parents' general wish to evoke positive rather than negative emotions in young children (Cole and Tan, 2006).

### Cultural patterns for norms about negative emotions

Our second goal was to investigate the cultural differences in regard to the frequency rate of negative displays. The hypothesis that American storybook characters would display more negative powerful and less negative powerless emotions compared to the characters in Turkish and Romanian storybooks was confirmed. The proportion of these two negative types of emotion was balanced in American storybooks. On the other hand, negative powerful emotions were displayed very infrequently in the Turkish and Romanian storybooks and the proportion of negative powerless displays were substantially higher than those in the American books. This result points to the importance of differentiating between types of negative emotions for cultural comparisons. Such a distinction was already effective for emotion studies of gender differences (Fischer and Manstead, 2000), and it may also be effective for cultural studies as interdependent cultures do restrict negative powerful emotions. An increasing number of emotion socialization studies have also begun to differentiate between specific types of negative emotions (US: Morris et al., 2011; Turkey: Corapci et al., 2012; India: Raval and Martini (2009); Nepal: Cole and Tan, 2006). These studies point to culturally distinct uses of emotion socialization practices in response to negative powerful emotions (e.g., anger) and powerless negative emotions (e.g., sadness; see Friedlmeier et al., 2011, in press). In general, the pattern of the findings from the current study is consistent with previous research such that negative powerful emotions are less acceptable in group-oriented cultures compared to negative powerless emotions because the former threaten the harmony while the latter elicit support, and these norms are also indicated in these storybooks. This pattern of findings supports the predictions drawn from the self-construal framework (Markus and Kitayama, 2010) and emotion competence models (Chan et al., 2009). The avoidance of displaying negative powerful emotions was especially obvious in Romanian storybooks as 15% of those displays even occurred when alone (while less than 1% in the American and Turkish books) and not in social situations. Overall, the display of negative powerful emotions was very low in the Romanian and Turkish books.

### Cultural pattern of emotion expression intensity

Our third goal was to investigate the cultural differences in emotion expression intensity. The hypothesis (3) that US children's books would display higher intensity of positive and negative powerful emotion expressions compared to Romanian and Turkish books was confirmed partially. We found support for the expected cultural difference in positive emotion expressions but not for negative powerful emotion expressions. Furthermore, the assumption that the intensity of negative powerless emotion would be higher in Romanian and Turkish books than in American books was rejected. Contrary to our expectations, the intensity of negative powerless emotions showed the same cultural pattern as for positive emotions: American storybook characters displayed powerless emotions such as fear and sadness more intensely compared to the characters in Romanian and Turkish storybooks, although the frequency of powerless emotions was lower as reported above. Overall, the results of the present study rather point to stronger emotion expression regardless of emotion valence. The general higher expression intensity in American storybook characters is in line with cross-cultural differences reported in display rules and emotion expression studies with adult (Van Hemert et al., 2007; Matsumoto et al., 2008) and child participants (Cole et al., 2002; Novin et al., 2009; Wilson et al., 2012). The findings of the present study strengthen the assumption that the self-orientation in Western, individualistic cultures emphasizes open communication of emotions to support the assertion of self (e.g., Gottman et al., 1997). These emotions indicate inner states of autonomous individuals who rely on themselves to achieve goals and whose states are not readily understood by others without the individual's expressing them (Markus and Kitayama, 2001).

### Impact of social context on emotion displays

The fourth goal of the present study was to investigate the differential emotion expression frequency and intensity as a function of the social group. We expected higher ingroup-outgroup differentiation for the Romanian and Turkish storybooks compared to American storybooks. This hypothesis was confirmed for positive emotion intensity regarding the Turkish and American storybooks but not for the Romanian ones. As expected, the characters in the Turkish storybook pictures were depicted as showing positive emotions more intensely to ingroup compared to outgroup members, whereas American books did not make such a distinction. Unexpectedly, Romanian books were similar to the American books regarding the social context effect on the positive emotions. The hypothesis was only partly confirmed for frequency of negative powerful emotions. Negative powerful emotions were overwhelmingly displayed to outgroup members in Romanian compared to American books. This pattern fits with the norm to avoid displaying such emotions to ingroup members as such emotions threaten the group harmony. Opposite to the hypothesis, all negative powerful emotions of the Turkish books occurred in ingroup contexts.

Another interesting result pertained to the role of social context on negative powerless emotions. Specifically, emotions such as sadness and fear were displayed in higher intensity toward ingroup members in all three countries and were shown about half of the time toward ingroup vs. outgroup members. The depiction of story characters as expressing powerless emotions more intensely to familiar relatives, best friends, or teachers rather than to strangers makes sense given the greater expectation of receiving emotional support and assistance from ingroup members. Our finding suggests that this expectation cuts across all cultural groups. This cultural similarity with respect to the ingroup-outgroup distinction also supports previous findings (Matsumoto et al., 2008) that in spite of a stronger norm of consistency Americans moderated and adapted the expression of negative emotions along contextual features.

### Different emotion norms in Romanian and Turkish books?

The results about the function of the context point to differences between Romanian and Turkish books. Either this difference refers to the lack of statistical power based on low frequency rates (e.g., low number of negative powerful emotions) or the emotion norms between Romania and Turkey are different. We assumed commonalities between Romanian and Turkish emotion norms. Although they share some norms and history, both countries differ in their political and economic development over the last 20 years. Since the breakdown of the communist system in the 90s, Romania became a democratic state, joined the EU, and started to be more oriented toward Western Europe in the last 10 years compared to Turkey. This was also noticeable in the fact that more of the popular Romanian books were translated books from Western countries compared to Turkish books. At the same time, Romania is still considered a rather traditional than a modern society according to value research like the European Value Survey (see M. Friedlmeier and Gavreliuc, 2013). Obedience and conformity are still important child-rearing goals, and these goals may keep alive the cultural norm to avoid strong emotion expressions, even negative powerless emotions, within the family. The analysis here suggests that negative emotions, especially negative powerful emotions, are less valued in Romanian compared to Turkish books. Most of the negative powerful emotions were displayed to outgroup members, and those emotions occurred also often when the protagonist was alone, which devalues such display toward others, especially familiar persons.

### General cultural features of storybooks beyond emotions

This book analysis brought some interesting insights beyond the hypotheses testing: the books showed differences in some contextual features that can be interpreted as indicators for differences in general cultural norms. Romanian and Turkish books presented more group contexts, i.e., more figures showing emotions were displayed on average on one page. However, this difference was limited to the codable figures. We did not count figures that were also present but in the background (too small to code) or did not show any emotion expression. The emotion displays in the Romanian and Turkish books were presented more often toward outgroup members. The emphasis on (out) groups may indicate a stronger collectivist perspective, group-orientation, and interdependence model. The American books presented emotions mostly in private and familiar settings. These characteristics match well with the norms of individualism combined with family orientation which is characteristic for US culture (see Bellah et al., 1985).

### Emotions in storybooks reflecting cultural emotion norms

We selected the books by popularity. It is noteworthy to report that children's books with a storyline, many pictures of figures, and a low amount of text, may not be a universal feature in itself. When we started to look for children's books, we also looked into the option to have authors and illustrators from the same country. This search showed that such types of books are not common in Romania and Turkey. When we switched to the popularity criterion, we could find many translated books, mostly from English, French, or German. As these types of children's books were popular and became more popular over the last 10 years, this form of medium can be seen as a sign of cultural change in the Eastern European cultures. At the same time, such translated books sometimes can be an informative source about cultural differences, because the publisher may change the images in the books, and sometimes even the story changes.

Authors and illustrators of books have different intentions and may not consciously tell stories and present drawings based on culturally shared emotion norms. Nevertheless, we can assume that emotion norms are reflected in and transmitted through media like children's storybooks, and the results of this study underscore that culture-specific emotion norms regarding salience and intensity of emotion expressions can be found in such media. It was an interesting result that many self-conscious emotions like guilt, embarrassment, shame, and pride nearly did not show up at all and were represented at a very low level. Given the fact that these emotions develop in the preschool period (Lewis, 2008), book authors may assume that they cannot yet be grasped so easily by children, so they avoid exposing them to these types of emotions.

### Limitations

A global rating of the emotion type and the intensity of expression was used to generate the main data for hypothesis testing. Similar to Tsai et al. (2007), we aimed to use FACS for coding emotion expressions. However, we met some limitations. Tsai et al. (2007) used FACS but they restricted the coding to limited features of positive emotions. As we expanded to negative emotions, the coding became more complicated. FACS is restricted to six basic emotions, and we aimed to include a wider range of emotions. Illustrators may not necessarily follow the emotion expression codes of FACS. Therefore, we decided to integrate the context information into coding of the type of emotion. The interrater agreement across coders form the different countries were satisfactory, but improvement of coding is desired for future studies. The results for the negative powerful emotions are limited due to the low number of occurrences, especially in Romanian and Turkish books. More books need to be coded to get more detailed insight into this type of emotion. Although the number of books was small, the number of coded images was rather high, and as we sampled the most popular books, we can assume that the audience of the books is widespread in the respective countries.

## Conclusions and outlook

This study shows that young children in Romania and Turkey are less exposed to intensive emotion expressions and they are less exposed to negative powerful emotions in popular children's storybooks. The media exposure for young children may occur when looking at those books on their own, but overwhelmingly they will experience these books through the presentation of a caregiver. Therefore, it is important to know how a parent reads to the child, i.e., how much they emphasize emotions and which ones. Some researchers investigated how mothers read wordless picture books and tell stories to young children, and cultural comparisons mostly refer to US-China comparisons (Doan and Wang, 2010; Tao et al., 2013). Early childhood teachers play also an important role (Denham and Bassett, 2012) for emotion socialization of young children, and no cultural comparison has been published so far. It remains a task for a future study to analyze how elaborately parents and teachers engage in the reading activity (i.e., the richness of detail they add to the story and pictures).

Knowledge about cultural difference in emotion norms is an important requisite to derive more precise hypotheses about cultural differences when studying emotion socialization with children and caregivers from a developmental perspective. Although such emotion norms are related to individualism/collectivism or independent/interdependent models of self (Markus and Kitayama, 2001; Tsai et al., 2006; Mesquita and Albert, 2007), they are not completely determined by such general norms. Insights into culturally shared emotion norms lead to better predictions of emotion socialization practices by caregivers for future cross-cultural developmental studies. For example, caregivers believing in the importance of strong excitement as ideal affect may encourage such expression of positive emotions in their children, whereas caregivers guided by contentment as ideal affect may even minimize the same displayed positive emotions of their children. Similar culture-specific emotion socialization strategies can be expected based on different norms regarding the negative emotions.

## Author contributions

Wolfgang Friedlmeier and Briana Vander Wege started the idea for this project. Briana Vander Wege developed the coding system and coded all the variables for all books. Mayra L. Sánchez González contributed to the coding system and coded most of the variables for all books. Briana Vander Wege and Mayra L. Sánchez González wrote a first draft of the article that was then revised by Wolfgang Friedlmeier. Wolfgang Friedlmeier supervised the writing of the theoretical and method part, wrote the result and discussion section of the first draft and wrote the complete revised version. Linda M. Mihalca coded all Romanian and most of the American books and she contributed to later drafts of the article. Erica Goodrich was mostly responsible for data handling and data analysis and also contributed to later drafts of the manuscript. Feyza Corapci was responsible for the selection and coding of the Turkish books and contributed to later drafts.

### Conflict of interest statement

The authors declare that the research was conducted in the absence of any commercial or financial relationships that could be construed as a potential conflict of interest.

## Footnotes

^1^Different ethnic groups within US may favor different storybooks. Some popular Hispanic-American books are available in both languages (English and Spanish). Therefore, we decided to focus on European-American children's storybooks.
